# Rural Primary Care Workforce Development in Serious Illness Care: The VA Palliative Scholars Program

**DOI:** 10.1093/geroni/igaf122.3169

**Published:** 2025-12-31

**Authors:** Sumi Misra, Katharina Echt

**Affiliations:** Vanderbilt University Medical Center, Nashville, Tennessee, United States; Emory University, Atlanta, Georgia, United States

## Abstract

Evidence demonstrates the quality of advanced and serious illness care is substantively improved by integration of Palliative Medicine practices. The demand for trained palliative care professionals remains unmet with pronounced national workforce gaps in rural communities. We developed and implemented an interdisciplinary primary care team training initiative that leverages the experience of well-established palliative care teams to provide an immersive virtual training experience that blends live case-based learning embedded in clinical encounters with interdisciplinary role appreciation, teamwork, prognostication, breaking bad news, pain, symptom management, team dynamics, and value-based goals of care discussions. Between May and September 2024 six multidisciplinary cohorts of palliative scholars (N = 32) completed the 3-day practicum. In-depth pre-post practicum metrics measured change in knowledge, attitudes, perceived skill, comfort, confidence with recognizing, addressing, and holding end-of-life conversations with patients and families. The scholars post evaluations demonstrated learners’ experienced significant gains in knowledge of and comfort with providing essential palliative and supportive care in the primary care setting. Knowledge increased by 85%, 100% indicated having gained valuable communication skills to discuss advance care planning and goals of care, 90% ranked their experience as highly satisfactory, and 100% indicated intention to recommend the practicum to others. Scholars reported feeling empowered to continue learning as champions for Palliative Care principles at their primarily rural settings of care. Initial evaluation of the Palliative Scholars model supports it viability as one effective solution to expanding the primary palliative care competency of our workforce to meet the needs of older adults with life-limiting illness.

